# *Lingguizhugan* decoction improves non-alcoholic fatty liver disease by altering insulin resistance and lipid metabolism related genes: a whole trancriptome study by RNA-Seq

**DOI:** 10.18632/oncotarget.19734

**Published:** 2017-07-28

**Authors:** Mingzhe Zhu, Shijun Hao, Tao Liu, Lili Yang, Peiyong Zheng, Li Zhang, Guang Ji

**Affiliations:** ^1^ Institute of Digestive Diseases, China-Canada Center of Research for Digestive Diseases, Shanghai University of Traditional Chinese Medicine, Shanghai, China; ^2^ Public Health College, Shanghai University of Traditional Chinese Medicine, Shanghai, China

**Keywords:** Lingguizhugan decoction, gene expression, non-alcoholic fatty liver disease, RNA-Seq

## Abstract

Lingguizhugan decoction, a classic traditional Chinese medicine formula, has been used to treat non-alcoholic fatty liver disease (NAFLD), however, the underlying mechanisms remains unclear. In the present study, we compared the phenotype of the normal rats (fed with chow diet), high-fat-diet (HFD) induced NAFLD rats and *Lingguizhugan* decoction (LGZG, comprises four Chinese herbs: *Poria*, *Ramulus Cinnamomi*, *Rhizoma Atractylodis Macrocephalae*, and *Radix Glycyrrhizae*.) intervened rats, and detected whole genome gene expression by RNA-Seq. Our results demonstrated that LGZG decoction attenuated phenotypic characteristics of NAFLD rats. RNA-Seq data analysis revealed that gene expression profiles exerted differential patterns between different groups. 2690 (1445 up-regulated, 1245 down-regulated) genes in NAFLD versus (*vs*) normal group, 69 (16 up-regulated, 53 down-regulated) genes in LGZG *vs* NAFLD group, and 42 overlapped (12 up- regulated, 30 down-regulated) genes between NAFLD*vs* normal group and LGZG *vs* NAFLD group were identified as differentially expressed. GO, pathway enrichment and PPI networks analysis of the overlapped genes revealed that LGZG decoction might attenuate NAFLD possibly by affecting insulin resistance and lipid metabolism related pathways (e.g., PI3K-Akt, AMPK). Differentially expressed genes involved in these pathways such as Pik3r1, Foxo1, Foxo3, Scd1, Col3a1 and Fn1 might be candidate targets for treating NAFLD.

## INTRODUCTION

Non-alcoholic fatty liver disease (NAFLD) is one of the leading causes of chronic liver diseases around the world, which is characterized by accumulation of fat deposits in the liver resulting from causes other than alcohol abuse [1, 2]. Prevalence of NAFLD has been increasing due to the modern sedentary and food-abundant lifestyle, which is 20-30% in western countries and 15-20% in China [3, 4]. Importantly, NAFLD is one of the most dangerous complications that can lead to severe liver pathologies, including fibrosis, cirrhosis and hepatocellular carcinoma [5, 6]. In addition, accumulating evidence indicates that the risks of NAFLD extend beyond the liver and are associated with a range of chronic diseases such as cardiovascular disease, chronic kidney disease and type 2 diabetes mellitus [7, 8].

Development of NAFLD is related to several factors such as age, gender, ethnicity, presence of sleep apnea and endocrine system disorders (e.g., hypothyroidism, hypopituitarism, hypogonadism, and polycystic ovarian syndrome). Prevalence of NAFLD increases with age and is more common among males aged 45-65 years. It is also spreading rapidly among children along with obesity epidemics [9]. Thus, NAFLD has become a major global health problem and brought heavy burden to patients and society.

The mechanisms involved in the development of NAFLD are still under debate, although several hypotheses have been proposed [10–12]. Studies have revealed that various signaling pathways such as nuclear factor κB (NF-κB), AMP-activated protein kinase (AMPK), Toll-like receptor (TLR) and phosphatidylinositol 3- kinase/protein kinase B (PI3K/Akt) are involved in the progress of NAFLD. These signaling pathways are interconnected to form a network, blocking any one of them may not be effective to prevent or treat the NAFLD [13]. Actually, there is no established standard therapy for NAFLD. International guidelines recommend that lifestyle changes as the main strategies suggested for NAFLD patients. However, pharmacological intervention are needed when the disease are progressive or serious. Recently, many complementary therapies, especially herbal medicines have been introduced to treat NAFLD [14].

Traditional Chinese medicine has been used for the treatment of suboptimal health from the perspective of predictive, preventive and personalized medicine (PPPM), which may be a cost-effective way for preventing chronic diseases [15]. Of note, a number of traditional Chinese medicines have been evaluated in treating NAFLD in the past decades. *Lingguizhugan* decoction is an ancient Chinese herbal formula from a classic book of traditional Chinese medicine titled Jingui Yaolue. Recently, *Lingguizhugan* decoction has been applied to prevent metabolic syndrome and may exhibit effects in alleviating obesity, hyperglycemia, hyperlipidemia, hypertension and hepatic injury [16]. It is reported that the modified *Lingguizhugan* decoction combined with short-term very low calorie diets could be safely implemented for steady glycemic control in newly diagnosed type 2 diabetes mellitus patients [17].Other researchers revealed that *Lingguizhugan* decoction combined calorie restriction could reduce the body weight, fasting plasma glucose and insulin resistance index in high fat diet feeding rat [18]. Our previous study also showed that *Lingguizhugan* decoction could attenuate HFD induced NAFLD, as measured by body weight, liver injury and hepatic triglyceride (TG) [19]. However, the underlying mechanisms of *Lingguizhugan* decoction on NAFLD remain unclear.

To enhance our understanding of the mechanisms under the efficacy of *Lingguizhugan* decoction, we applied HFD induced NAFLD rats and firstly detected hepatic whole genome gene expression profile by RNA-Seq analysis. The present study may shed new lights on understanding the scientific connotation of traditional Chinese medicine therapeutic principle in NAFLD and provide more options for treating NAFLD and candidate targets for clinical therapy of NAFLD in the future.

## RESULTS

### Phenotypic characteristics of the rats

**Figure 1:**
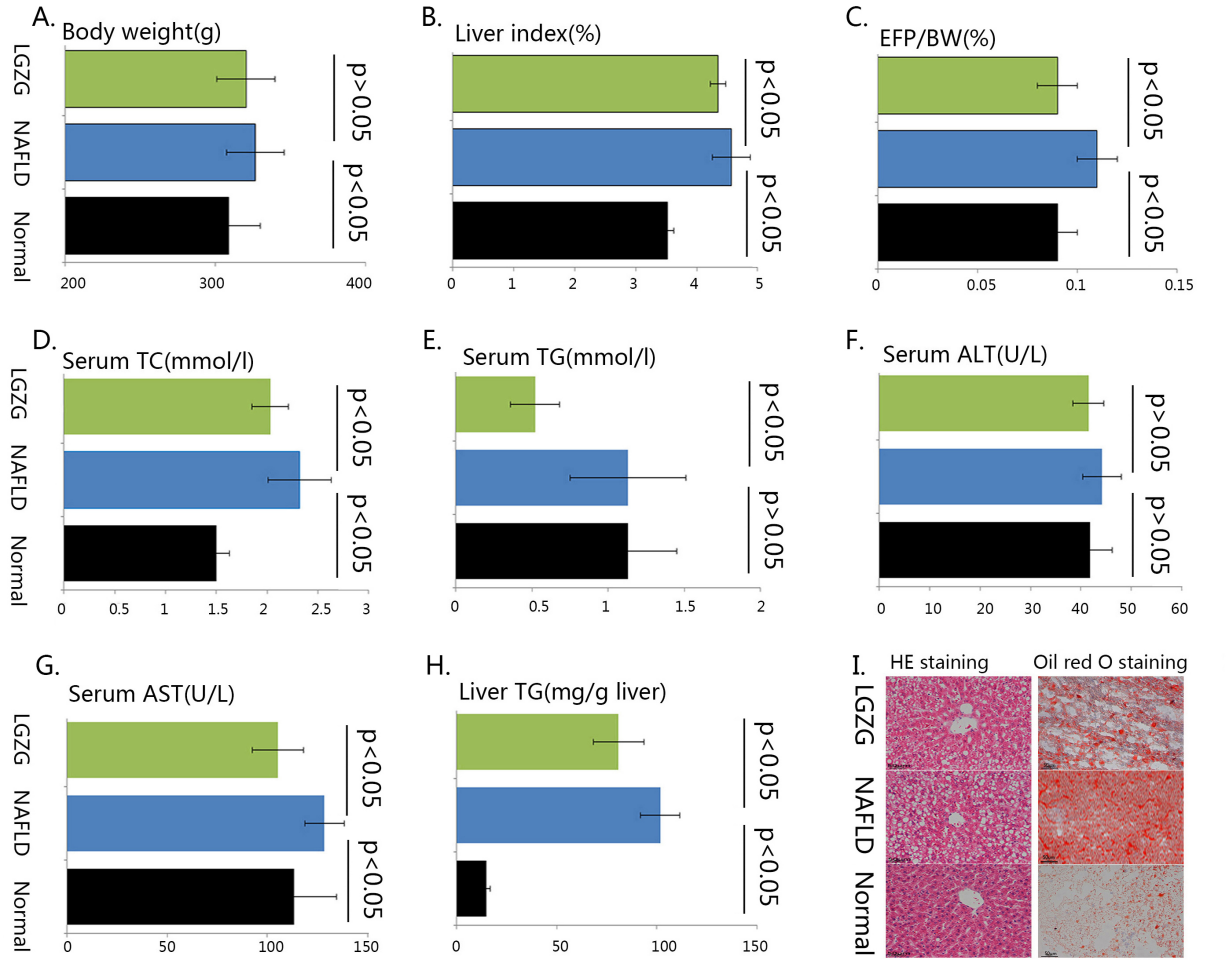
The phenotypic characteristics of the rats Male C57Bl/6 mice were either fed chow diet (Normal, n=7), HFD (n=7) or HFD with *Lingguizhugan* decoction administrated (n=7) for 4 weeks The rats were sacrificed, and liver tissue and serum were collected. Body weight was recorded **(A)**, liver index **(B)**, Epididymal Fat Pad-Body Weight Ratio **(C)** were calculated. Serum TC **(D)**, TG **(E)**, ALT **(F)**, AST **(G)** were analyzed, liver TG content **(H)** was detected, and liver sections were stained with both H&E and oil Red O **(I)** ( image magnification ×200). Data were present as mean ± SD.Green bars, *lingguizhugan* decoction intervened group; blue bars, HFD induced NAFLD group; black bars, normal group. Two compared groups were marked with a line and statistical information was given as *p*<0.05 or *p*>0.05.

HFD feeding induced steatosis, which is the typical characteristic of NAFLD in liver sections as demonstrated by hematoxylin and eosin (H&E) staining and Oil Red O staining (Figure 1I), significantly increased hepatic TG (Figure 1H) in NAFLD rats was consistent with the histological alteration. The bodyweight (Figure 1A), liver index (Figure 1B), Epididymal Fat Pad-Body Weight Ratio (EFP/BW, Figure 1C), serum total cholesterol (TC, Figure 1D), serum aspartate aminotransferase (AST, Figure 1G) were also dramatically increased in NAFLD rats, but serum TG (Figure 1E) and almandine aminotransferase (ALT, Figure 1F) showed no significantly difference between groups. After 4-week *Lingguizhugan* decoction treatment, steatosis was attenuated (Figure 1I) and hepatic TG content were significantly decreased (Figure 1H). Liver index, EFP/BW ratio (Figure 1C), serum TC (Figure 1D), TG (Figure 1E) and AST (Figure 1G) were also markedly decreased upon *Lingguizhugan* decoction intervention, whereas body weight (Figure 1A) and serum ALT (Figure 1F) did not respond to the intervention.

### Differentially expressed gene profiles

Using the cutoff false discovery rate<0.05 and fold change>1.2 (up- regulated) or<0.83(down-regulated), we identified 2690 (1445 up- regulated, 1245 down-regulated) differentially expressed genes in NAFLD group comparing to normal group. Interestingly, we observed *Lingguizhugan* decoction altered gene expression profiles and identified 69 (16 up-regulated, 53 down-regulated) differentially expressed genes in *Lingguizhugan* decoction intervention group (LGZG) in comparison to NAFLD group. We also identified 42 overlapped (12 up- regulated, 30 down- regulated) genes between NAFLD *vs* normal group and LGZG *vs* NAFLD group. The differentially expressed genes in NAFLD *vs* normal and LGZG *vs* NAFLD were listed in supplementary file 1. The overlapped differentially expressed genesbetween NAFLD *vs* normal group and LGZG *vs* NAFLD group were listed in Table 1 .

**Table 1:** The overlapped differentially expressed genes list

Gene_symbol	Fold change NAFLDVs normal	FDR NAFLDVs normal	Fold change LGZGvs NAFLD	FDR LGZGvs NAFLD
Actn4	1.220	0.024	0.797	0.029
Ahr	1.378	0.019	0.663	0.046
Alas1	1.228	0.017	0.644	0.004
Apon	0.740	0.004	1.330	0.031
Avpr1a	1.341	0.023	0.622	0.040
Bcl2l11	1.708	0.017	0.556	0.028
Col3a1	1.295	0.015	0.734	0.006
Ech1	0.645	0.002	1.412	0.026
Fads2	0.523	0.004	0.727	0.041
Fn1	1.295	0.016	0.764	0.016
Foxo1	1.645	0.022	0.494	0.032
Foxo3	1.562	0.033	0.426	0.012
Fzd1	1.271	0.030	0.520	0.025
Gstt3	1.281	0.018	1.356	0.047
Hfe2	0.658	0.007	1.637	0.028
Hspa8	0.696	0.001	0.791	0.027
Igfbp7	1.282	0.017	0.723	0.040
Insig1	0.698	0.016	0.400	0.003
Irf2bp2	1.422	0.022	0.542	0.037
Lpin1	1.518	0.016	0.406	0.000
Lrp1	1.203	0.020	0.823	0.016
Mafb	1.478	0.015	0.533	0.025
Osgin1	0.427	0.004	2.360	0.004
Pik3r1	1.292	0.027	0.535	0.003
Ppp1r3c	2.385	0.002	0.361	0.000
Rapgef5	1.502	0.030	0.490	0.047
Rasgef1b	2.073	0.012	0.429	0.010
Rnf186	0.139	0.008	4.726	0.033
Scd1	1.866	0.011	0.561	0.023
Slc25a32	1.323	0.025	0.440	0.000
Slc38a2	1.540	0.005	0.483	0.000
Tmem2	1.669	0.015	0.621	0.049
Tp53inp1	2.122	0.016	0.402	0.019
Tsc22d3	1.464	0.016	0.582	0.018
Zfp189	2.368	0.007	0.366	0.010

**Figure 2:**
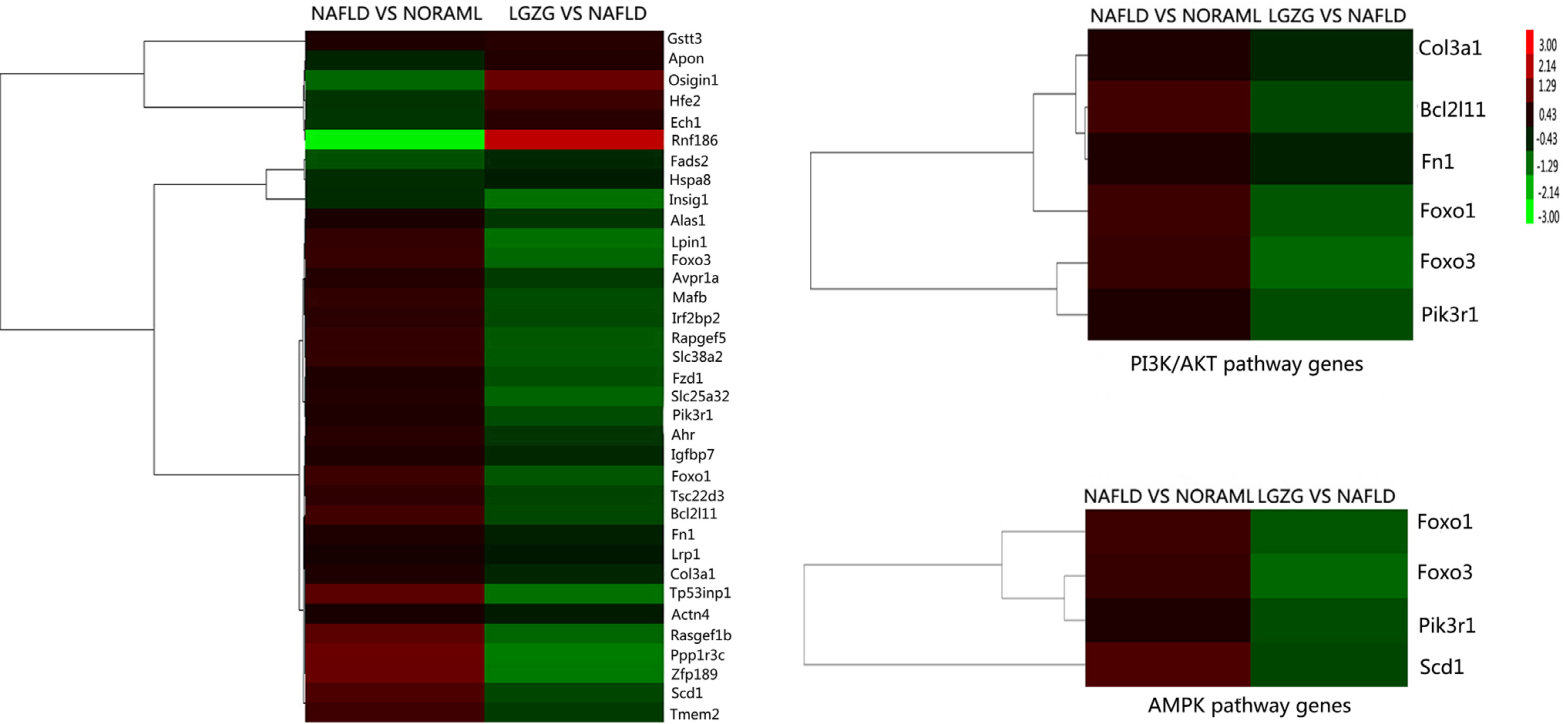
Hierarchical cluster for the overlap differentially expressed genes All the relevant genes were grouped by hierarchical clustering based on expression values (log2 ratios) across all the samples Samples are displayed in columns and genes in rows. Gene expression is represented as a color, with brighter red for higher values and brighter green for lower values.

Furthermore, we performed hierarchical cluster analysis for the overlapped genes and observed distinctly different patterns of gene expression. As shown in Figure 2, most of the differentially expressed genes in NAFLD *vs* normal group had the opposite expression patterns in LGZG *vs* NAFLD group, e.g. Pik3r1, Forkhead O transcription factors 1 (Foxo1), stearoyl CoA desaturase 1 (Scd1) were up-regulated in NAFLD rats, whereas down-regulated in LGZG group. Additionally, genes enriched in PI3K/Akt and AMPK pathways also exhibited different expression patterns.

### Gene annotation and functional analysis

**Figure 3:**
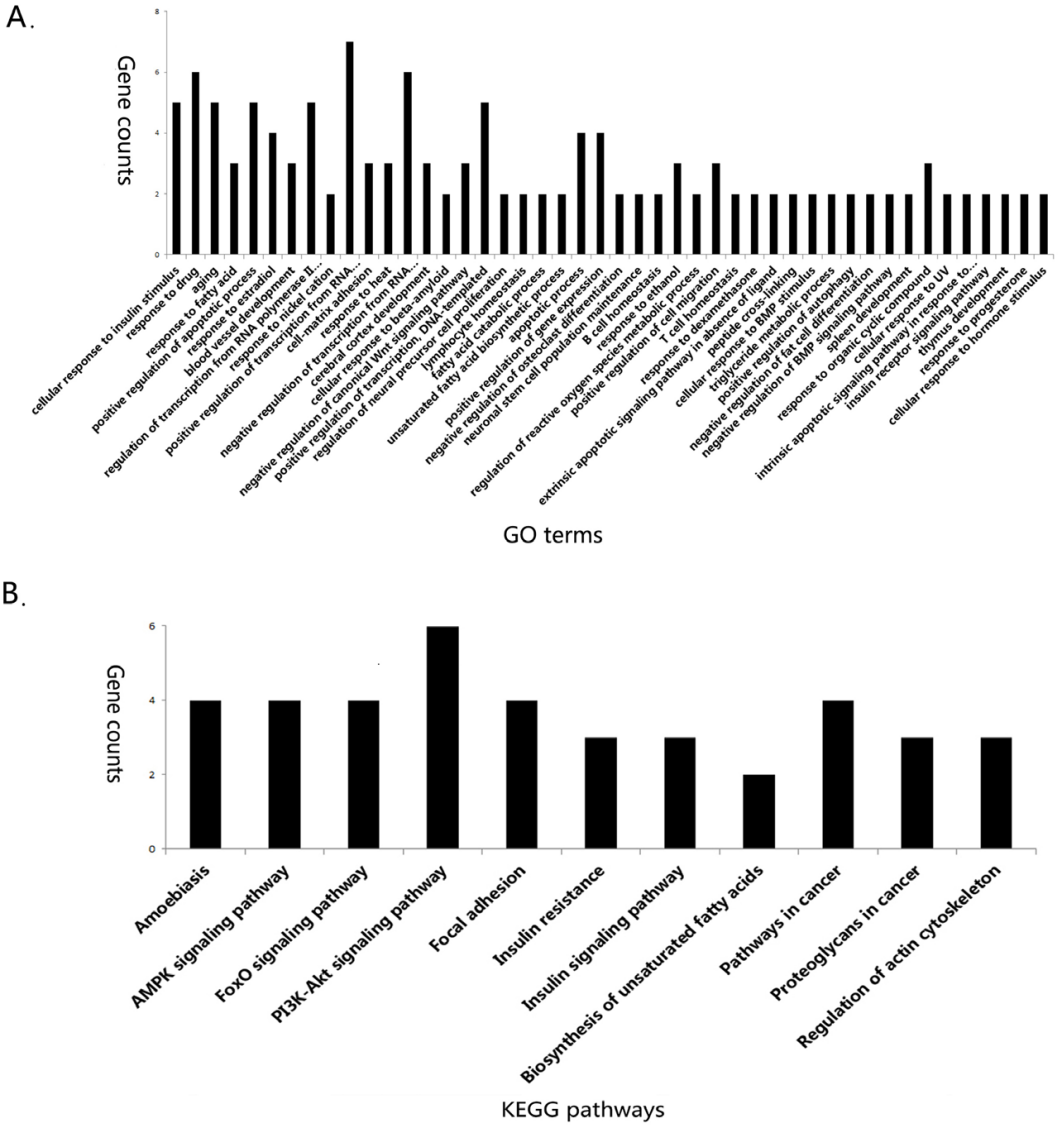
GO and KEGG pathway enrichment GO and KEGG pathway enrichment were analyzed by David 6.8 GO (biological process) terms enriched for overlapped differentially expressed genes **(A)** KEGG pathway enrichment for overlapped differentially expressed genes **(B)**.

To examine whether there was recognizable biological relevance to the gene expression patterns, further gene annotation and functional analysis were performed for the 42 overlapped differentially expressed genes. Using David 6.8, we noticed that 35 were known and 7 were unknown genes. Functional category of the overlapped genes was classified into Gene Ontology (GO) terms such as cellular response to insulin stimulus, response to drug, response to fatty acid and positive regulation of apoptotic process (Figure 3A). The detailed information for GO terms of overlapped genes was listed in supplementary file 2. Pathway enrichment analysis revealed that 21 overlapped genes were enriched in Kyoto Encyclopedia of Genes and Genomes (KEGG) signaling pathways such as AMPK signaling pathway, PI3K-Akt signaling pathway and Foxo signaling pathway (Figure 3B). For instance, Foxo1, Foxo3, Pik3r1 and Scd1 were enriched in AMPK signaling pathway, Bcl2l11, Col3a1, Fn1, Foxo1, Foxo3 and Pik3r1 were enriched in PI3K/Akt signaling pathway. Of note, Foxo1, Foxo3, Pik3r1, Scd1, Bcl2l11, Col3a1 and Fn1 were up-regulated in NAFLD group, which were significantly decreased by *Lingguizhugan* decoction. The detailed information for the pathway enrichment was listed in Table 2. Gene annotation, GO and KEGG pathway analysis all revealed that the main differentially expressed genes altered by *Lingguizhugan* decoction were mainly focused on insulin resistance and lipid metabolism related functions.

**Table 2:** The overlapped differentially expressed genes pathway enrichment

KEGG pathway name	Genes enriched in pathway	P Value
Amoebiasis	FN1, Col3a1 Actn4, Pik3r1	0.002
AMPK signaling pathway	Foxo1, Foxo3, Scd1, Pik3r1	0.003
FoxO signaling pathway	Foxo1, Foxo3, Bcl2l11, Pik3r1	0.004
PI3K-Akt signaling pathway	Bcl2l11, Col3a1, Fn1, Foxo1, Foxo3, and Pik3r1	0.008
Focal adhesion	Fn1, Col3a1, Actn4, Pik3r1	0.014
Insulin resistance	Foxo1, Ppp1r3c, Pik3r1	0.029
Insulin signaling pathway	Foxo1, Ppp1r3c, Pik3r1	0.045
Biosynthesis of unsaturated fatty acids	Scd1, Fads2	0.069
Pathways in cancer	Foxo1, Fn1, Fzd1, Pik3r1	0.070
Proteoglycans in cancer	Fn1, Fzd1, Pik3r1	0.085
Regulation of actin cytoskeleton	Fn1, Actn4, Pik3r1	0.098

### Protein-protein interaction (PPI) network for the overlapped genes

**Figure 4:**
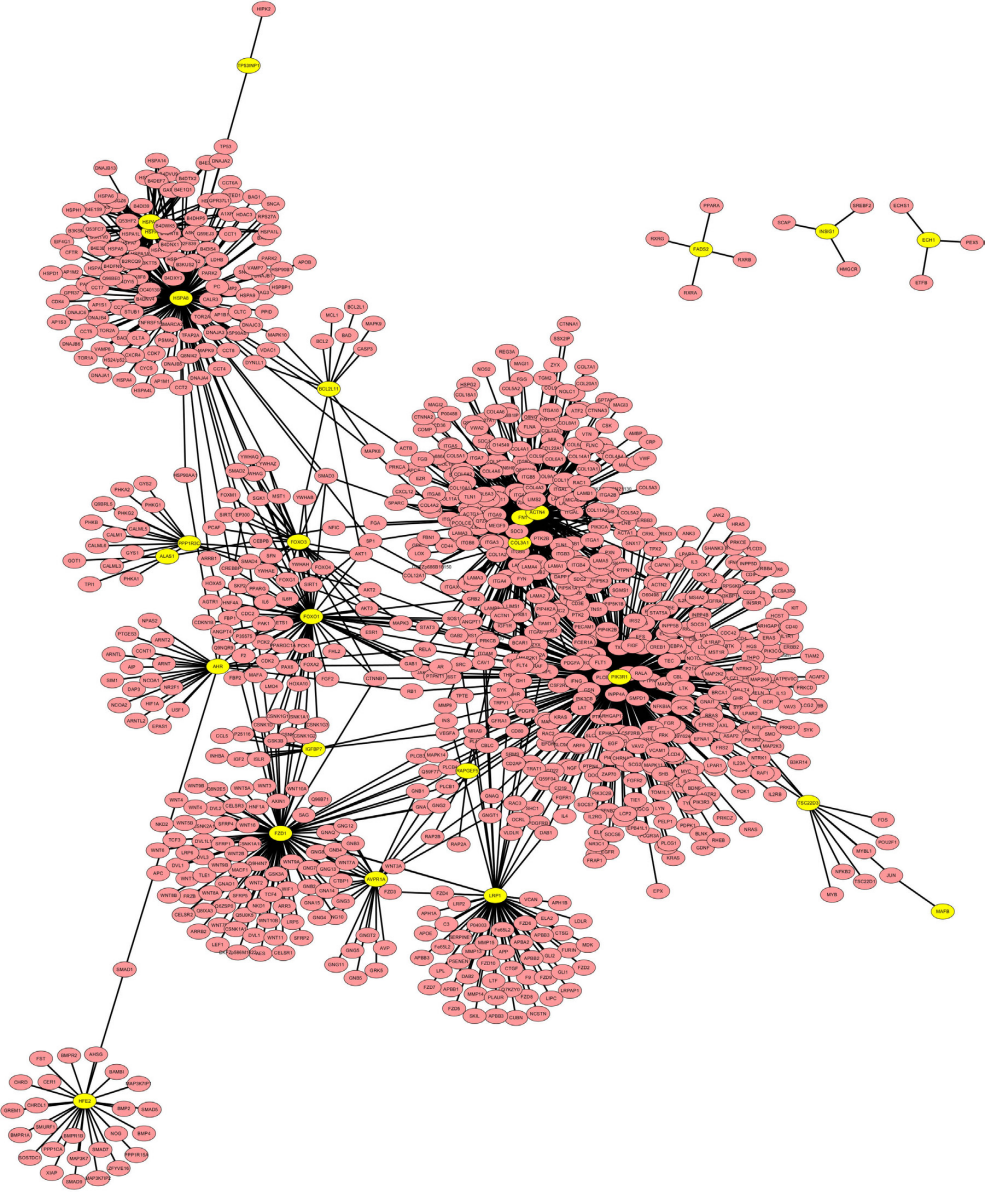
PPI network for the overlapped differentially expressed genes The yellow nodes represent overlapped differentially expressed genes encoding proteins, which are interacted with the predicted proteins (blue nodes).

PPI network of the overlapped differentially expressed genes was constructed by Cytoscape based on BioGRID database. Hub proteins indicated that the connectivity among the nodes was tightly established. As shown in Figure 4, we observed 23 differentially expressed genes encoding proteins were involved in the network such as Foxo1, Foxo3, PIK3R1, FN1, BCL2L11and COL3A1, of which, PIK3R1 was the most important hub protein.

## DISCUSSION

In the present study, applying RNA-seq analysis, we observed that *Lingguizhugan* decoction could improve the phenotypic characteristics of NAFLD and alter a batch of gene expression. By literature retrieval, we found most of the identified differentially expressed genes were related to NAFLD. Furthermore, using the combination of gene annotation, GO, KEGG pathway and PPI network analysis, we noticed that most of the genes altered by *Lingguizhugan* decoction were insulin resistance and lipid metabolism related genes.

Insulin resistance is pivotal for the progression of NAFLD [20]. Insulin signal transduction involves a cascade of factors, including insulin receptor desensitization, insulin receptor substrate (IRS), PI3K, and Foxo activated gene transcriptional profiling [21]. PI3K pathway plays an important role in the development of insulin resistance. PI3K belongs to the class 1a 3-kinases, which exist as heterodimers, consisting of a regulatory subunit (p85) and a catalytic subunit (p110). The abundance of p85 and p110 can alter PI3K activity. Accumulating evidence has shown that increased expression of PI3K p85 contributes to the pathogenesis of insulin resistance [22]. Xu D, et al [23] has reported PI3K p85 was significant higher in NAFLD patients compared with the control patients, which is in agreement with our present observation. Interestingly, PI3K p85 was significantly decreased by *Lingguizhugan* decoction, indicating that *Lingguizhugan* decoction might regulate the function of PI3K p85 to enhance PI3K activity, alleviate insulin resistance and improve NAFLD. FOXOs play a critical role in the integration of hormonal and nutritional signals for metabolic control. As a downstream mediator of insulin signaling, the activity of FOXOs is attenuated by PI3K/Akt [24]. FOXO1 is a direct transcriptional regulator of gluconeogenesis, and has profound effects on hepatic insulin resistance and lipid metabolism. FOXO3 is required for antioxidant responses and autophagy, its expression alters in liver diseases. A number of studies suggest that inactivation of FOXOs may block insulin resistance and decrease the risk of mortality from diabetes mellitus [25]. However, relatively less is known about FOXOs in NAFLD and controversial data were found. Valenti L, et al [26] reported that FOXO1 expression and activity are increased in patients with nonalcoholic steatohepatitis (NASH). In contrast, Pan X, et al [27] observed FOXOs play a salutary role in protecting mice against the diet induced fatty liver disease. In the present study, we observed Foxo1 and Foxo3 were up-regulated in NAFLD rats and remarkably reduced by *Lingguizhugan* decoction treatment. However, only through future investigations can safely and effectively develop FOXOs into viable clinical treatments for NAFLD.

Lipid metabolism alteration is another important factor for the progression of NAFLD. AMPK, is a heterologous trimeric protein kinase (composing of a catalytic α subunit and regulatory β, γ subunits), which is a central regulator of cellular energy balance and metabolic control, plays an important role in hepatic lipid metabolism [28]. Activation of AMPK directly suppresses activity of sterol regulatory element- binding protein-1c and -2 (SREBP-1c, SREBP-2), leading to decreased SREBP-1c target gene expression, including acetyl-CoA carboxylase 1 (ACC1), fatty acid synthase (FAS), and Scd1 transcription and translation, resulting in reduced lipid accumulation and improved liver condition [29]. We observed that *Lingguizhugan* decoction altered AMPK signaling pathway. For instance, gene Prkab1 encoding AMPKβ1was reduced in NAFLD rats and increased (1.5 fold) by *Lingguizhugan* decoction treatment, which deserved to be further investigation. Literature searching revealed that the main functional subunit of AMPK is AMPKα. Although rare data was reported for the correlation of AMPKβ1with NAFLD, it has been recorded that genetic deletion of the AMPK β1 subunit in mice displayed enhanced adipose tissue macrophage inflammation and hepatic insulin resistance [30]. Another study found that metformin and salicylate, which interacted directly at the AMPKβ1 drug-binding site, could reduce lipogenesis and enhance insulin sensitivity in mice [31].

Scd1 is a rate-limiting enzyme catalyzing the synthesis of monounsaturated fatty acids, which are major components of tissue lipids. Scd1 has been shown to be a crucial factor in lipid metabolism as a critical control point regulating hepatic lipid synthesis and β-oxidation [32].The genetic deletion of Scd1 protects against the development of fatty liver and insulin resistance in mice. Besides, antisense nucleotide inhibitors against hepatic Scd1 prevent HFD induced hepatic steatosis, and Scd1 inhibitors are claimed to be new treatments for NASH [33]. Our findings in the present study were consistent with these data, indicating *Lingguizhugan* decoction might decrease Scd1 and prevent the progress of NAFLD. Further investigation on the mechanisms of Scd1 regulation may help finding new drugs for NAFLD treatment.

Moreover, we also noticed some other genes such as Col3a1 and Fn1 were significantly down-regulated by *Lingguizhugan* decoction. Col3a1 transcripts into collagen alpha-1(III) chain protein, which is a precursor to collagen III, while Fn1 encodes glycoprotein fibronectin, the ligand of extracellular matrix components. These two genes are actively involved into the fibrosis process. Of note, Zhang X, et al [34] has reported that dioscin exhibited potent effects against liver fibrosis through modulation of Col3a1 expression. Chen G, et al [35] found that gossypol ameliorated liver fibrosis and reduced Col3a1and Fn1 levels. Bai Q, et al [36] reported that acetaminophen hepatotoxic metabolite, increased cellular mRNA expression of Col3a1 and induced liver fibrosis. All these data suggested that Col3a1and Fn1 play an important role in the progression of NAFLD and *Lingguizhugan* decoction might down- regulate Col3a1and Fn1.

Although there currently remains no approved pharmacological intervention for NAFLD, a series agents are studied to target associated comorbidities with varying degrees of success. Among them, Orlistat that prevents enteric lipid absorption is targeting obesity, and acts as a reversible lipase inhibitor [37]; Thiazolidinediones, the PPARγ agonists, are proved to be effective in enhancing insulin sensitivity [38]; The lipid-lowering agent statin is mostly used in treating dyslipidemia and mainly regulating cholesterol synthesis [39]; Obeticholic acid, a synthetic variant of chenodeoxycholic acid, is a potent activator of the farnesoid X nuclear receptor, and has been confirmed to improve NASH pathology [40]. As a formula that composed of natural products, it is reasonable that *Lingguizhugan* decoction was associated with insulin resistance and lipid metabolism.

In summary, *Lingguizhugan* decoction attenuated HFD induced NAFLD possibly by altering insulin resistance and lipid metabolism related pathways (PI3K-Akt, AMPK, etc). Differentially expressed genes involved in these pathways such as Pik3r1, Foxo1, Foxo3, Scd1, Col3a1 and Fn1 might be candidate targets for NAFLD therapy. It is a study of bioinformatics in exploring unknown mechanisms of natural compounds, and the results could infer valuable information for further studies, which might develop new patterns for new drug discovery. Although most of our data were consistent with other studies, several limitations of the present study should be noted. Firstly, the progress of NAFLD may be associated with age and gender. In the present study, the differentially expressed genes were mined from five-weeks-old male rats. Further investigations are needed to validate the candidate targets in groups with different ages and genders. Secondly, we do not know what extent of the altered genes expression acts on protein functions due to posttranslational modifications of proteins such as phosphorylation, ubiquitination, acetylation, lipidation, and glycosylation [41]. Further great efforts should be taken to evaluate protein functions encoding by these identified genes, which may shed new lights on novel treatments for NAFLD.

## MATERIALS AND METHODS

### Preparation of *Lingguizhugan* decoction

*Lingguizhugan* decoction comprises: *Poria* (main chemicals containing polysaccharides, triterpenes, etc), *Ramulus Cinnamomi* (main chemicals containing cinnamaldehyde, cinnamic acid, etc), *Rhizoma Atractylodis Macrocephalae* (main chemicals containing atractylone, polysaccharides, etc), and *Radix Glycyrrhizae* (main chemicals containing glycyrrhizinic acid, licoflavone, etc). The ratio of the four herbs was 2:1.5:1:1. All herbs were provided by Longhua Hospital affiliated to Shanghai University of Traditional Chinese Medicine. Herbal decoction was prepared as previously described [42].

### Animals and diets

Five- week- old male Wistar rats (130 g ± 10 g) were obtained from Shanghai SLAC Laboratory Animal CO. LTD, China, and maintained in temperature and humidity-controlled room. Twenty one rats were randomly divided into three groups: normal group, fed with chow diet; NAFLD group, fed with HFD (88% chow diet, 10% lard and 2% cholesterol); LGZG group, fed with HFD and administered with *Lingguizhugan* decoction at a dose of 10 mL/kg/d

Animals were weighed and sacrificed after 4-week intervention. Blood samples were harvested from the abdominal aorta, centrifuged at 3000 × g for 10 min at 4 °C and serum was collected. Liver tissue was quickly removed, rinsed with 0.9 % sodium chloride solution and weighed. Two pieces of liver tissue (1.0 cm×1.0 cm×0.2 cm) from the identical lobe and position were obtained and then fixed in 10% neutral-buffered formalin. The other liver tissue was stored at−80°C after snap frozen in liquid nitrogen. All animal procedures were approved by the Animal Experiment Ethics Committee of Shanghai University of Traditional Chinese Medicine

### Biochemical analysis

Serum almandine aminotransferase (ALT), AST, TG, TC and liver TG were assayed using commercial kits (obtained from Nanjing Jiancheng Bioengineering Institute, Nanjing, China) according to the manufacturers' instructions.

### Histopathological examination

Liver samples were fixed in 10% formalin for 48 hours, embedded in paraffin, sectioned to 4 μm thickness, stained with H&E, and frozen samples were cut into 8 μm sections and stained with Oil Red O, all the samples were examined under a light microscope at 200×magnification.

### RNA-Seq detection

Total RNA were isolated from liver tissues (7samples per group) using TRIzol reagent (Invitrogen, USA). RNA quality was verified using Agilent 2100 Bio-analyzer (Agilent Technologies, Santa Clara, CA). cDNA libraries preparation were subsequently performed with reagents supplied in Illumina’s TruSeq RNA sample preparation kit v2 according to the manufacturer’s instructions. The final purified libraries were evaluated using BioAnalyzer 2100 automated electrophoresis system, quantified and sequenced on the HiSeq 2000 according to Illumina’s standard sequencing protocol.

### RNA-Seq data analysis

To obtain high-quality clean data for downstream analysis, the low quality reads and reads containing adapters or ploy-N in raw data were removed. Clean reads were aligned with the rat genome (Ensembl RGSC3.4) using TopHat v2.1.1. The reads number mapped to each gene were counted by HTSeq 0.7.2. Fragments Per Kilobase of transcript sequence per millions base pairs sequenced (FPKM) was used to determine the transcription abundance of each gene. Differential expression of three groups (seven biological replicates per group) was analyzed using DESeq 2. False discovery rate<0.05 and fold change >1.2 or <0.83 were used as criteria for significantly differentially expressed genes.

Hierarchical cluster analysis was performed using Cluster 3.0 to define differential gene expression patterns. GO and KEGG functional pathways were analyzed by DAVID 6.8 (https://david.ncifcrf.gov/) for differentially expressed genes. Furthermore, PPI network was screened based on BioGRID database using Cytoscape software (http://cytoscape.org/). In the network, nodes represent proteins and edges represent interactions between two proteins.

### Statistical analysis

Data were expressed as mean ±SD and were analyzed by one-way analysis of variance (ANOVA) by SPSS v18.0 software. *P* value less than 0.05 was considered statistically different.

## SUPPLEMENTARY MATERIALS TABLES







## Abbreviations

NAFLD: non-alcoholic fatty liver disease
HFD: high-fat-diet
LGZG: *Lingguizhugan* decoction
NF-κB: nuclear factor κB
AMPK: AMP-activated protein kinas
TLR: Toll-like receptor
PI3K/Akt: phosphatidylinositol 3- kinase/protein kinase B
H&E: hematoxylin and eosin
EFP/BW: Epididymal Fat Pad-Body Weight Ratio
AST: aspartate aminotransferase
ALT: almandine aminotransferase
Foxo1: Forkhead O transcription factors 1
Scd1: stearoyl CoA desaturase 1
GO: Gene Ontology
KEGG: Genes and Genomes
NASH: non-alcoholic steatohepatitis
SREBP-1c: sterol regulatory element- binding protein-1c and -2
ACC1: acetyl-CoA carboxylase 1
FAS: fatty acid synthase

## References

[1] Ray K (2013). NAFLD-the next global epidemic. Nat Rev Gastroenterol Hepatol.

[2] Williams T (2015). Metabolic syndrome: nonalcoholic fatty liver disease. FP Essent.

[3] Ahmed M (2015). Non-alcoholic fatty liver disease in 2015. World J Hepatol.

[4] Farrell GC, Wong VW, Chitturi S (2013). NAFLD in Asia--as common and important as in the West. Nat Rev Gastroenterol Hepatol.

[5] Hardy T, Oakley F, Anstee QM, Day CP (2016). Nonalcoholic fatty liver disease: pathogenesis and disease spectrum. Annu Rev Pathol.

[6] Fusillo S, Rudolph B (2015). Nonalcoholic fatty liver disease. Pediatr Rev.

[7] Karim MF, Al-Mahtab M, Rahman S, Debnath CR (2015). Non-alcoholic fatty liver disease (NAFLD)--a review. Mymensingh Med J.

[8] Mikolasevic I, Milic S, Turk Wensveen T, Grgic I, Jakopcic I, Stimac D, Wensveen F, Orlic L (2016). Nonalcoholic fatty liver disease-A multisystem disease?. World J Gastroenterol.

[9] Moghaddasifar I, Lankarani KB, Moosazadeh M, Afshari M, Ghaemi A, Aliramezany M, Afsar Gharebagh R, Malary M (2016). Prevalence of non-alcoholic fatty liver disease and its related factors in Iran. Int J Organ Transplant Med.

[10] Day CP, James OF (1998). Steatohepatitis: a tale of two “hits”?. Gastroenterology.

[11] He X, Ji G, Jia W, Li H (2016). Gut microbiota and nonalcoholic fatty liver disease: insights on mechanism and application of metabolomics. Int J Mol Sci.

[12] Pan F, Liao N, Zheng Y, Wang Y, Gao Y, Wang S, Jiang Y, Liu X (2015). Intrahepatic transplantation of adipose-derived stem cells attenuates the progression of non-alcoholic fatty liver disease in rats. Mol Med Rep.

[13] Lu Zeng, Tang Wai J, Yin Jin J, Zhou Bei J (2014). Signal transductions and nonalcoholic fatty liver: a mini-review. Int J Clin Exp Med.

[14] Liu ZL, Xie LZ, Zhu J, Li GQ, Grant SJ, Liu JP (2013). Herbal medicines for fatty liver diseases. Cochrane Database Syst Rev.

[15] Wang W, Russell A, Yan Y, Global Health Epidemiology Reference Group (GHERG) (2014). Traditional Chinese medicine and new concepts of predictive, preventive and personalized medicine in diagnosis and treatment of suboptimal health. EPMA J.

[16] Yao L, Wei J, Shi S, Guo K, Wang X, Wang Q, Chen D, Li W (2017). Modified *Lingguizhugan* decoction incorporated with dietary restriction and exercise ameliorates hyperglycemia, hyperlipidemia and hypertension in a rat model of the metabolic syndrome. BMC Complement Altern Med.

[17] Chen DS, Ke B, Huang YJ, Meng J, Zhang JJ, Chen ZX, Michalsen A, Qin J (2011). Effects of the modified Linggui Zhugan Decoction combined with short-term very low calorie diets on glycemic control in newly diagnosed type 2 diabetics. J Tradit Chin Med.

[18] Wang YY, Jin MH, Ke B, Li SH, Shen YZ, Zhai JY, Chen CY, Qin J (2013). [Effects of linggui zhugan decoction combined calorie restriction on the insulin resistance of model rats and mechanisms research]. [Article in Chinese]. Chin J Integr Med.

[19] Liu T, Yang LL, Zhang L, Song HY, Li DF, Ji G (2012). Comparative study on the effects of different therapeutic methods in preventing and treating nonalcoholic fatty liver in rats. Zhong Xi Yi Jie He Xue Bao.

[20] Kitade H, Chen G, Ni Y, Ota T (2017). Nonalcoholic fatty liver disease and insulin resistance: new insights and potential new treatments. Nutrients.

[21] Guo S (2014). Insulin signaling, resistance, and the metabolic syndrome: insights from mouse models intodisease mechanisms. J Endocrinol.

[22] Draznin B (2006). Molecular mechanisms of insulin resistance: serine phosphorylation of insulin receptor substrate-1 and increased expression of p85α. Diabetes.

[23] Xu D, Huang XD, Yuan JP, Wu J, Fan Y, Luo HS, Yang YH (2011). Impaired activation of phosphatidylinositol 3-kinase by leptin is a novel mechanism of hepatic leptin resistance in NAFLD. Hepatogastroenterology.

[24] Rui L (2014). Energy metabolism in the liver. Compr Physiol.

[25] Maiese K (2015). FoxO transcription factors and regenerative pathways in diabetes mellitus. Curr Neurovasc Res.

[26] Valenti L, Rametta R, Dongiovanni P, Maggioni M, Fracanzani AL Zappa M, Lattuada E, Roviaro G, Fargion S (2008). Increased expression and activity of the transcription factor FOXO1 in nonalcoholic steatohepatitis. Diabetes.

[27] Pan X, Zhang Y, Kim HG, Liangpunsakul S, Dong XC (2017). FOXO transcription factors protect against the diet- induced fatty liver disease. Sci Rep.

[28] Jeong KJ, Kim GW, Chung SH (2014). AMP-activated protein kinase: An emerging target for ginseng. J Ginseng Res.

[29] Zhu X, Bian H, Gao X (2016). The potential mechanisms of berberine in the treatmentnt of nonalcoholic fatty liver disease. Molecules.

[30] Galic S, Fullerton MD, Schertzer JD, Sikkema S, Marcinko K, Walkley CR, Izon D, Honeyman J, Chen ZP, van Denderen BJ, Kemp BE, Steinberg GR (2011). Hematopoietic AMPK β1 reduces mouse adipose tissue macrophage inflammation and insulin resistance in obesity. J Clin Invest.

[31] Ford RJ, Fullerton MD, Pinkosky SL, Day EA, Scott JW, Oakhill JS, Bujak AL, Smith BK, Crane JD, Blümer RM, Marcinko K K, Kemp BE, Gerstein HC (2015). Metformin and salicylate synergistically activate liver AMPK, inhibit lipogenesis and improve insulin sensitivity. Biochem J.

[32] Flowers MT, Ntambi JM (2008). Role of stearoyl-coenzyme A desaturase in regulating lipid metabolism. Curr Opin Lipidol.

[33] Uto Y (2016). Recent progress in the discovery and development of stearoyl CoA desaturase inhibitors. Chem Phys Lipids.

[34] Zhang X, Han X, Yin L, Xu L, Qi Y, Xu Y, Sun H, Lin Y, Liu K, Peng J (2015). Potent effects of dioscin against liver fibrosis. Sci Rep.

[35] Chen G, Wang R, Chen H, Wu L, Ge RS, Wang Y (2016). Gossypol ameliorates liver in diabetic rats induced by high-fat diet and streptozocin. Life Sci.

[36] Bai Q, Yan H, Sheng Y, Jin Y, Shi L, Ji L, Wang Z (2017). Long-term acetaminophen treatment induced liver fibrosis in mice and the involvement of Egr-1. Toxicology.

[37] Harrison SA, Fecht W, Brunt EM, Neuschwander-Tetri BA (2009). Orlistat for overweight subjects with nonalcoholic steatohepatitis: a randomized, prospective trial. Hepatology.

[38] Boettcher E, Csako G, Pucino F, Wesley R, Loomba R (2012). Meta-analysis: pioglitazone improves liver histology and fibrosis in patients with non-alcoholic steatohepatitis. Aliment Pharmacol Ther.

[39] Chatrath H, Vuppalanchi R, Chalasani N (2012). Dyslipidemia in patients with nonalcoholic fatty liver disease. Semin Liver Dis.

[40] Neuschwander-Tetri BA, Loomba R, Sanyal AJ, Lavine JE, Van Natta ML, Abdelmalek MF, Chalasani N, Dasarathy S, Diehl AM, Hameed B, Kowdley KV, McCullough A, Terrault N (2015). Farnesoid X nuclear receptor ligand obeticholic acid for non-cirrhotic, non-alcoholic steatohepatitis (FLINT): a multicentre, randomised, placebo-controlled trial. Lancet.

[41] WangY Adua E, Russell AC, Roberts P, Ge S, Zeng Q, Wang W (2016). Glycomics and its application potential in precision medicine. Sci Suppl.

[42] Liu T, Yang LL, Zou L, Li DF, Wen HZ, Zheng PY, Xing LJ, Song HY, Tang XD, Ji G (2013). Chinese medicine formula lingguizhugan decoction improves beta-oxidation and metabolism of fatty Acid in high-fat-diet-induced rat model of fatty liver disease. Evid Based Complement Alternat Med.

