# Supplementary material for: Lingguizhugan decoction improves non-alcoholic fatty liver disease by altering insulin resistance and lipid metabolism related genes: a whole trancriptome study by RNA-Seq
Source: Oncotarget. 2017 Jul 28;8(47):82621–31. doi: 10.18632/oncotarget.19734 (PMC5669915; doi:10.18632/oncotarget.19734)
Supplement: Supplementary file 1 [file oncotarget-08-82621-s001.pdf]

Lingguizhugan decoction improves non-alcoholic fatty liver disease by altering insulin resistance and lipid metabolism related genes: a whole trancriptome study by RNA-Seq

SUPPLEMENTARY MATERIALS

Supplementary Table 1: The differentially expressed genes (DEGs) among groups

See Supplementary File 1

Supplementary Table 2: The detailed information for GO terms of overlapped genes

See Supplementary File 2
